# Cocaine and Its Rational Antidote

**Published:** 1900-09

**Authors:** G. Lenox Curtis

**Affiliations:** New York City


### Abstracts and Translations.

### COCAINE AND ITS RATIONAL ANTIDOTE.[1]

BY G. LENOX CURTIS, M.D., NEW YORK CITY.

In the majority of cases in which cocaine is used some excitement either pleasant or unpleasant is manifested. The pulse becomes rapid, the breathing quick and deep, followed by headache, dryness of the throat, pallor of the face, nausea, and coldness of the extremities, accompanied by a tingling sensation; the skin becomes clammy, and often great beads of perspiration form; the eyes grow glassy and the pupils dilate. When a large amount of the drug has been ingested, convulsions, either tonic or clonic, may occur, or collapse may follow. Death is due to gradual cessation of respiration.

Cocaine is a stimulant to the central nervous system. It increases cerebral activity and endurance of fatigue. For generations the natives of Peru and Bolivia ate cocoa-leaves as a stimulant, and their soldiers were provided with them to chew when making forced marches. Scientific experiments prove that more work can be done after taking cocaine. The heart's action is accelerated by cocaine owing to the direct action of the drug on the cardiac muscle and stimulation of the cardiac sympathetic. Paralysis of the vagus, as in belladonna poisoning, cannot account for the increased activity, for stimulation of the vagus in a case of cocaine poisoning slows the heart, showing that the latter nerve has not been deprived of its function. At first the blood-vessels are much contracted, which, with the rapid pulse-rate, causes a marked rise in the blood-pressure. The cause of the arterial contraction is stimulation of the vasomotor centre. Subsequently the blood-pressure falls from peripheral vasomotor paralysis.

The local effect of the drug is due to paralysis of the termini of some of the afferent nerves, particularly those conveying impressions of pain and touch, but the temperature sense does not seem to be affected. Cocaine acts best on mucous membranes. In the

[1] Read before the Union Dental Meeting, Richmond, Va., May 10, 1900.

nose it paralyzes the sense of smell as well as sensation, but it has very little effect, if any, on the healthy skin. Schleich's method of infiltration anæsthesia is probably the most satisfactory. I have found that a weak solution of cocaine is especially applicable in work on the mucous membrane, but in operations on the deeper tissues, and in bone work, the stronger solution is more effective. I therefore use from a ten per cent. to a saturated solution. The great advantage gained by employing solutions of high strength is economy of time in the operation, which to a busy practitioner is important. In from one to two minutes after the injection the surgeon can proceed and the operation be completed by the time a weaker solution would have taken effect.

The most successful surgeons of to-day aim to consume the least possible time in operating, and thus lessen shock.

The opportunities for the use of cocaine are numerous. It is effective in major as well as in minor operations. If more operators would follow Schleich's example, much of the discomfort and danger of general anæsthesia would be averted. I predict that the time will come when ether and chloroform will be held in reserve as emergency drugs, and that cocaine, or some other local anæsthetic, will supersede them. I am able to do fully ninety per cent. of my work with cocaine. The principal objection to it is its toxic effect; if that can be overcome by an antidote, surgery will forge ahead and many major operations will become minor ones.

Cushing says, "Cocaine is a protoplasmic poison. It destroys the protoplasm of nerve-end organs, hence explains its local anæsthetic action. When a solution of cocaine comes in contact with other organs, it destroys their vitality. Ciliated epithelial cells, leucocytes, and spermatozoa become motionless. Cortical nerve-cells lose their excitability. Many of the invertebrates are killed by even a short exposure to cocaine. Movements of protoplasm in plants are also retarded or entirely suppressed by this poison." This doubtless accounts to a greater or less degree for the general languor that usually follows the use of cocaine. In continued daily operations where cocaine is employed the strength and energy of the patient decline, and often a morbid condition exists.

A rational antidote cannot be expected to prevent protoplasmic poisoning or destruction. Operations are not usually done on the same patient every day, hence nature may be safely permitted to look out for local ill effects, which, to say the least, are never serious.

A successful antidote must antagonize the paralyzant effect of cocaine upon the heart, the blood-vessels, respiration, etc. It should comprise in its physiological action the merits of digitalis or strophanthus, belladonna, ergot, calabar bean, etc. In its effect upon the circulation and respiration, volasem, which is an extract of violet, resembles the principal action of these drugs. Its effect is manifested so quickly and surely that with it any required strength and amount of cocaine can be safely used. Volasem neutralizes the general toxic effect of cocaine, but does not interfere with its local effect. It stimulates the heart's action and contracts the arterioles. It stimulates the respiration and raises the blood-pressure. When administered immediately before cocaine is employed, it prevents the usual untoward symptoms by maintaining the respiratory and cardiac functions. I have found that where volasem was administered in five-drop doses, every hour, until twelve doses had been taken, no appreciable action was observed; but when fifteen drops were given every half hour for two hours, its action upon the heart and lungs was similar to the primary effect of cocaine, but none of the other cocaine symptoms were observed. I have also noticed with susceptible patients that ten drops would produce similar results within a minute or two. These cases respond quickly to cardiac stimulants, and have none of the usual cocaine after-effects. I found, however, that hypodermic injection of cocaine would immediately restore the equilibrium. Thus I am led to believe that these two drugs antidote each other.

To show the efficacy of volasem, I will relate some clinical experiences.

Mrs. A., aged forty, upon whom I had previously operated under cocaine, was to be operated upon again, this time for the removal of a tumor. When ready, I discovered I had no volasem, but concluded to proceed under a four per cent. solution of cocaine. I injected four drops and waited for its effect. In about two minutes the patient showed unmistakable toxic symptoms. Aromatic spirits of ammonia was quickly administered, and by the time her clothing was loosened alarming symptoms appeared. The patient being unconscious, hypodermic injections of digitalis, whiskey, and strychnine were given. Most of the extreme symptoms were manifested. Respirations had fallen to seven a minute; the radial and temporal pulse ceased, and the heart's action was scarcely perceptible. It required an hour's hard work to restore the patient, and it was several days before she was in a normal condition. Two weeks later I went on with the operation, first giving five drops of volasem and a minute later injecting thirty drops of a ten per cent. solution of cocaine into and about the tumor. I completed the operation in twenty minutes, the patient showing not the slightest effect of the cocaine. She expressed her astonishment at the virtue of the antidote.

Another phase of the toxic effect of cocaine and the quick action of volasem was recorded in my discussion of Dr. Foster's paper on cocaine poisoning, published in the *Dental Cosmos* for November, 1898. The patient was brought to me by his dentist on the eve of my summer vacation in 1898. As the case was urgent, I concluded to operate with the doctor's assistance. I prepared the volasem, but forgot to give it. I injected half a drachm of a saturated solution of cocaine. Within a few seconds the patient complained of a peculiar sensation pervading his entire body and a tingling in the extremities. He became unconscious, and was soon fighting like a demon. It was with great effort we prevented his doing us bodily harm, when suddenly toxic convulsions occurred. I turned to give him more volasem, when I discovered I had not given him any. Prying open the mouth, I poured the ten-drop dose down his throat. After the lapse of a minute the muscular rigidity relaxed, and within another minute restoration was complete. The patient stated he had no knowledge of what had happened. I finished the operation, and within an hour he went to his home, apparently none the worse for his experience.

